# Glomangiomyoma of the Knee: A Rare Juxtasynovial Presentation: Juxtasynovial glomangiomyoma in the joint capsule is a rare location for glomus tumors that should be considered in the differential diagnosis of hypervascular synovial-based tumors

**DOI:** 10.5334/jbsr.2051

**Published:** 2020-03-03

**Authors:** Nico Hustings, Filip Vanhoenacker, Adelard De Backer

**Affiliations:** 1 Sint-Lucas Hospital Ghent, BE; 2 Sint-Maarten Hospital Mechelen, BE

**Keywords:** glomus tumor, glomangiomyoma, knee, juxtasynovial tumor, MRI

## Abstract

Glomus tumors are benign tumors typically located in the subcutis or deep dermis of the subungual region of the fingers. Histologically, glomus tumors are divided into three subtypes, in descending order of frequency: solid glomus tumor, glomangioma and glomangiomyoma. We report a case of a symptomatic intracapsular juxtasynovial glomangiomyoma of the knee. To the best of our knowledge, this location of this uncommon histological subtype of glomus tumor has not been reported previously. Although the final diagnosis is made by histopathology, the radiologist should consider this rare lesion in the differential of highly vascularised synovial-based masses.

## Introduction

Glomus tumors are benign neoplasms arising from the glomus apparatus, which has a role in thermoregulation and is typically located in the dermis [1]. Glomus tumors are relatively rare, accounting for 1.6% of soft tissue tumors in the extremities [2]. Histologically, glomus tumors are subdivided into solid glomus tumor, glomangioma or glomangiomyoma with a frequency of 75%, 20% and 5% respectively [2]. Glomus tumors are typically subungually located. Extradigital locations, of which location around the knee joint is the most prevalent, are rarely seen. Glomus tumors adjacent to the joint capsule are very rare and – to the best of our knowledge – intracapsular juxtasynovial location has not yet been reported in the literature [1,2,3,4,5,6,7,8,9,10,11,12].

## Case Report

A 64-year-old woman presented with gradually increasing pain and reduced mobility of the right knee. Clinical examination showed limited motion of the knee and painful palpation in the medial and retropatellar aspect of the joint. There were no apparent skin changes, nor palpable masses.

MRI showed a 4 cm well-delineated solid mass within the medial part of the patellofemoral joint extending into the infrapatellar fat pad and limited by the medial collateral ligament (Figures A–D). The lesion was closely related to the underlying synovium of the joint space. There was no invasion into adjacent bone or subcutaneous soft tissues. The lesion was isointense to muscle on T1-weighted image (WI) and hyperintense on fat-suppressed (FS) proton density (PD) T2-WI. Tortuous feeding vessels and intense homogeneous enhancement was noted on fat-suppressed T1-WI after gadolinium contrast administration.

**Figures A–D:**
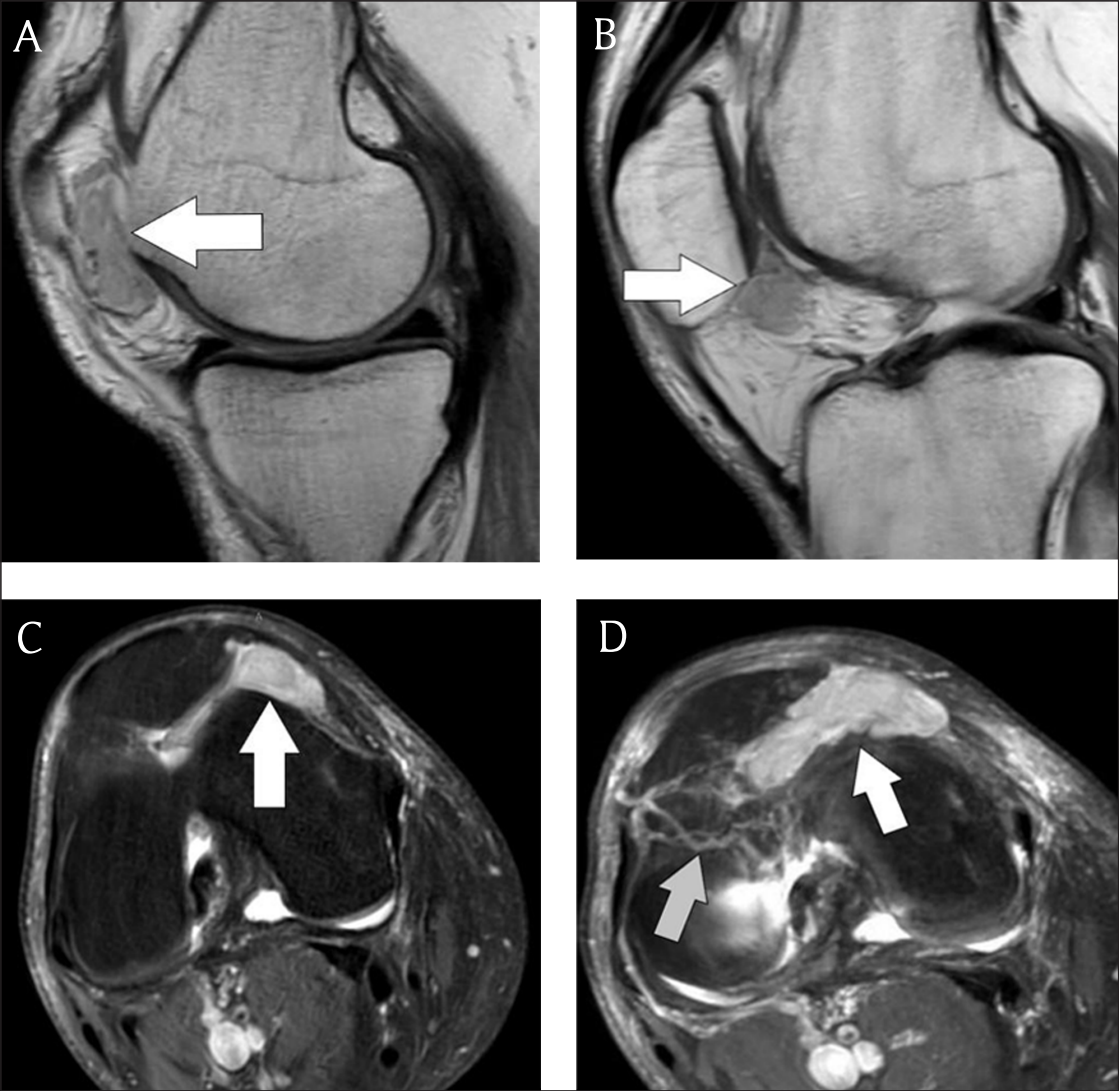
MRI imaging of the right knee. **A** and **B.** Sagittal T1-WI (2 adjacent slices) shows a well circumscribed solid mass (*white arrow*) in the infrapatellar fat pad that is isointense to muscle. The lesion shows a close relationship with the underlying synovium of the joint space. **C.** Axial fat-suppressed proton density T2-WI shows a hyperintense mass (*white arrow*) extending into the medial part of the patellofemoral joint that is limited by the medial collateral ligament. **D.** Axial fat-suppressed T1-WI after gadolinium contrast administration. Note vivid homogeneous contrast enhancement of the mass (*white arrow*) and the presence of tortuous feeding vessels (*grey arrow*).

Complete open surgical resection was performed and confirmed that the lesion was located inside the articular capsule and was attached to the underlying synovium. The postoperative follow-up was uneventful, and the patient was completely pain-free after eight weeks.

Histopathological examination of resection specimen revealed a proliferation of thick-walled blood vessels surrounded by a monotonous population of small cells with a round and regular nucleus. No cytonuclear atypia, atypical mitoses or other malignant characteristics were retained. Immunohistochemistery was strongly positive for smooth muscle actin (SMA) and weakly positive for synaptophysin. Markers for CD-34 and desmin were negative. Based on histological findings, a diagnosis of glomangiomyoma was made.

## Discussion

Glomus tumors are typically small, usually less than 1 cm, and comprise only 1.6% of soft tissue tumors in the extremities. Histologically, glomus tumors are subdivided into solid glomus tumor, glomangioma or glomangiomyoma with a frequency of 75%, 20% and 5% respectively. In the solid type, the glomus cells predominate. Glomus cells are small, uniform cells containing eosinophilic cytoplasm and rounded nuclei. The glomangioma type also contains glomus cells, but has a greater vascularity. The least frequent glomangiomyoma type demonstrates glomus cells, vascular channels and smooth muscle cells. Glomangiomyoma are positive on immunohistochemy tests for SMA, myosin, vimentin, and desmin [2].

Glomus tumors are usually located underneath the nail bed, but may infrequently occur at extradigital locations [3]. Extradigitally located glomus tumors are most commonly seen in the dermis of the knee. However, intracapsular glomus tumors with juxtasynovial location are very rare [1,2,3,4,5,6,7,8,9,10,11,12]. Two cases were reported of glomus tumors within the infrapatellar fat pad, but there was no close relationship with underlying synovium [4,5]. One case report by Damle et al. (2013) describes a glomus tumor which was attached to the synovium, but was located in the subcutis [6].

The typical presentation of subungual glomus tumors consists of a triad of paroxysmal pain, cold sensitivity and tenderness to palpation. The symptoms are disproportional to the small size of the tumor and may cause disuse syndromes due to the extreme pain [10]. However, the reported extradigital glomus tumors, in particular around the knee, usually do not present with typical clinical features of subungual glomus tumors [11].

MRI imaging is the preferred technique to localize and characterize glomus tumors. The lesion is seen as a solid, well-defined round or ovoid mass, and highly vascularised with prominent feeding vessels. The tumor is of low to intermediate signal on T1-WI, isointense compared to muscle. The lesion is of high signal on T2-WI and PD fat-suppressed WI. On contrast-enhanced T1-WI a marked and homogeneous enhancement is seen [12].

The differential consists of pigmented villonodular synovitis, tenosynovial giant cell tumor, melanoma, vascular malformation and other vascular neoplasms [12]. Pigmented villonodular synovitis and tenosynovial giant cell tumor may show a similar contrast-enhancement pattern but due to hemosiderin deposition, the lesion contains low-signal-intense foci on T1- and T2-WI, causing blooming artifacts on T2* sequences [13]. Synovial melanoma may show hyperintense foci on T1-WI due to the presence of melanin [14]. Vascular malformations involving the synovium show vascular channels with fluid-fluid levels, intermediate signal on T1-WI, high signal on T2-WI and diffuse contrast-enhancement. Often the lesions contain fatty components and the lesion may have both intra- and extraarticular extension [15].

Complete surgical resection of glomangiomyoma is the treatment of choice and results in prompt disappearance of symptoms. Other therapeutic options are sclerotherapy or embolization [11].

## Conclusion

In conclusion, glomangiomyoma juxtasynovially located in the joint capsule is an extremely rare manifestation of glomus tumors and should be included in the differential diagnosis of hypervascular synovial-based tumors. However, histopathology is needed for definitive diagnosis.
