# Correction: Glomangiomyoma of the Knee: A Rare Juxtasynovial Presentation

**DOI:** 10.5334/jbsr.2166

**Published:** 2020-06-01

**Authors:** Nico Hustings, Filip Vanhoenacker, Adelard De Backer

**Affiliations:** 1 Sint-Lucas Hospital Ghent, BE; 2 Sint-Maarten Hospital Mechelen, BE

**Keywords:** glomus tumor, glomangiomyoma, knee, juxtasynovial tumor, MRI

## Abstract

This article details a correction to the article: Hustings N, Vanhoenacker F, De Backer A. Glomangiomyoma of the Knee: A Rare Juxtasynovial Presentation: Juxtasynovial glomangiomyoma in the joint capsule is a rare location for glomus tumors that should be considered in the differential diagnosis of hypervascular synovial-based tumors. Journal of the Belgian Society of Radiology. 2020;104(1):12. DOI: http://doi.org/10.5334/jbsr.2051.

## Correction

The article, ‘Glomangiomyoma of the Knee: A Rare Juxtasynovial Presentation: Juxtasynovial glomangiomyoma in the joint capsule is a rare location for glomus tumors that should be considered in the differential diagnosis of hypervascular synovial-based tumors’ [1] was published with an incorrect title. The correct title is: Glomangiomyoma of the Knee: A Rare Juxtasynovial Presentation.
